# Human talkers change their voices to elicit specific trait percepts

**DOI:** 10.3758/s13423-023-02333-y

**Published:** 2023-07-28

**Authors:** Stella Guldner, Nadine Lavan, Clare Lally, Lisa Wittmann, Frauke Nees, Herta Flor, Carolyn McGettigan

**Affiliations:** 1 Department of Child and Adolescent Psychiatry and Psychotherapy, Central Institute of Mental Health, Medical Faculty Mannheim, Heidelberg University, Mannheim, Germany; 2 Institute of Cognitive and Clinical Neuroscience, Central Institute of Mental Health, Medical Faculty Mannheim, Heidelberg University, Mannheim, Germany; 3 Department of Psychology, Queen Mary University of London, London, UK; 4 Department of Speech, Hearing and Phonetic Sciences, University College London, London, UK; 5 Institute of Psychology, University of Regensburg, Regensburg, Germany; 6 Institute of Medical Psychology and Medical Sociology, University Medical Centre Schleswig Holstein, Kiel University, Kiel, Germany

**Keywords:** Voice production, Impressions, Self-presentation, Social interactions, Communication

## Abstract

The voice is a variable and dynamic social tool with functional relevance for self-presentation, for example, during a job interview or courtship. Talkers adjust their voices flexibly to their situational or social environment. Here, we investigated how effectively intentional voice modulations can evoke trait impressions in listeners (Experiment 1), whether these trait impressions are recognizable (Experiment 2), and whether they meaningfully influence social interactions (Experiment 3). We recorded 40 healthy adult speakers’ whilst speaking neutrally and whilst producing vocal expressions of six social traits (e.g., likeability, confidence). Multivariate ratings of 40 listeners showed that vocal modulations amplified specific trait percepts (Experiments 1 and 2), which could be explained by two principal components relating to perceived affiliation and competence. Moreover, vocal modulations increased the likelihood of listeners choosing the voice to be suitable for corresponding social goals (i.e., a confident rather than likeable voice to negotiate a promotion, Experiment 3). These results indicate that talkers modulate their voice along a common trait space for social navigation. Moreover, beyond reactive voice changes, vocal behaviour can be strategically used by talkers to communicate subtle information about themselves to listeners. These findings advance our understanding of non-verbal vocal behaviour for social communication.

**Supplementary Information:**

The online version contains supplementary material available at 10.3758/s13423-023-02333-y.

## Introduction

Beyond language, the sound of the human voice conveys information about a person’s identity (McGettigan, 2015), and vocal cues are actively used by listeners to judge a talker’s personality, emotions, physical attributes or intentions (Hellbernd & Sammler, 2016; Krauss et al., 2002; Kreiman & Sidtis, 2011; Pisanski et al., 2016; Puts et al., 2006; Sauter et al., 2010; Scherer, 1972). Even from short, neutral utterances (‘hello’) listeners make rapid social judgements about a talker (McAleer et al., 2014). These various social judgments can be summarized by a small number of dimensions, often referring to affiliation and competence – reflecting a common and modality-independent, two-dimensional social space (see Oosterhof & Todorov, 2008, for research on faces and Fiske et al., 2007, for social stereotypes). The affiliation dimension conveys information about a speaker’s likeability, warmth, trustworthiness or valence, whereas the competence dimension signals hierarchy or dominance. McAleer et al. (2014) extended this social trait space to voices (“the social voice space”), showing that social judgements based solely on a speaker’s voice can also be summarized by these two underlying dimensions. Although these impressions are rapidly formed and do not necessarily reflect reality, they guide future interactions (Cuddy et al., 2007; Olivola et al., 2014). Judgements from voices have been shown to be predictive of success in a job interview (Schroeder & Epley, 2015), election behaviour (Pavela Banai et al., 2017; Tigue et al., 2012), or preferences during romantic courtship (Feinberg et al., 2005).

However, talkers are also active agents in vocal communication. This means that talkers can, for example, construct their social identity and navigate their social landscape through linguistic variation (Eckert, 2012). Beyond linguistic variation, the human voice itself is a highly dynamic signal, with talkers being able to expertly control their larynx and the articulators of the upper respiratory tract and change their voice to suit the acoustic and/or communicative context (Lavan et al., 2019; Scott & McGettigan, 2016). Thus, the voice is a potentially important medium allowing someone to strategically manage social interactions and actively guide trait impressions from listeners via intentional vocal modulation. Indeed, previous research has already shown that natural within-person variability in the voice – based on randomly selected samples of natural speech – can affect how observers rate an individual’s personality on a range of traits (Lavan et al., 2021). However, the social trait space has never been explored in the context of *intentional* vocal behaviour. Here, there are two important questions to ask. First, can a person volitionally modulate the information in their voice to influence specific listener percepts and hence change their own location in social trait space? Hughes et al. (2014) provided partial evidence by showing that talkers were rated higher in dominance and intelligence when they intentionally tried to convey these traits, compared to speech in their ‘neutral’ voice. However, ratings of trait modulations in comparison to each other via multivariate ratings are needed to characterise specific movement on the social voice space more comprehensively. That is, when a talker aims to express likeability, do they only sound more likeable or do they also sound more confident? This allows us to determine the specificity and inter-relations of intentional voice modulations. Second, do vocal modulations have functional relevance for social interactions? Work on face perception shows that viewers prefer different images of a person for different scenarios (e.g., dating vs. political campaigning; Todorov & Porter, 2014), therefore deliberate changes in the voice should show similar functionality in situations where social outcomes can be influenced by how a person sounds.

### The present work

In three studies we aimed to examine how intentional vocal modulations can guide perception. Experiment 1 combined vocal production and perception tasks to test the sensitivity and specificity with which talkers can volitionally convey social traits to listeners. Specifically, we tested whether vocal modulations (in comparison to a talker’s neutral voice) would amplify perceptual ratings of the intended expressed trait in comparison to other traits. To align our findings with previous research, we further examined whether modulated voices would evoke changes in trait percepts (over and above the perception of the talker’s neutral voice) that could be explained by the two primary dimensions of the vocal trait space. In two additional perceptual studies, we then probed the replicability and generalisability of our findings: In Experiment 2, we tested whether listeners could selectively recognise an intended trait amongst other modulated voice samples using categorical labels (e.g., ‘likeable’, ‘hostile’). In Experiment 3, we then asked whether the evoked trait percepts in listeners would generalize from categorical trait labels (e.g., ‘likeable’) to functionally relevant social scenarios, by measuring whether listeners could selectively match modulated voices to trait-appropriate social goals (e.g., a likeable voice for a friendly situation).

## Experiment 1

### Introduction

In this first experiment, we tested the proposal that intentional voice modulations can induce specific changes in listeners’ impressions of a talker, using multivariate listener ratings of traits.

### Methods

#### Talkers

Forty native German talkers (age *M* = 22.08 years, *SD* = .27 years; 13 male) were recruited as a subgroup of a larger cohort from the European IMAGEN study (for a detailed description, see Schumann et al., 2010). We based the sample size on a previous study (Hughes et al., 2014), and post hoc power simulations for linear mixed-effects models using *simr* in *R* (Green & Macleod, 2016) showed a minimal achieved power of 0.96. All participants had normal or corrected-to-normal vision and normal hearing. All participants provided informed written consent prior to their participation and were reimbursed with a monetary reward for their participation (hourly rate: 10 €). This study was approved by the research ethics committee of the Medical Faculty Mannheim of Heidelberg University (2007-024-N-MA).

#### Vocal modulation task

All talkers were asked to read a list of sentences, while expressing six social traits: attractiveness, likeability, hostility, intelligence, confidence and dominance (for instructions and definitions of all social traits, see Online Supplementary Materials (OSM) S1). We additionally recorded non-modulated (neutral) voices as a baseline condition before recording modulated voice samples. The sentences used for all social traits (and the neutral condition) consisted of three semantically neutral German sentences (English translations: “*There are many bridges in Paris”* / “*Many flowers bloom in July”* / “*Bears eat a lot of honey”*), which were paired with each expressed trait.

**Fig. 1:**
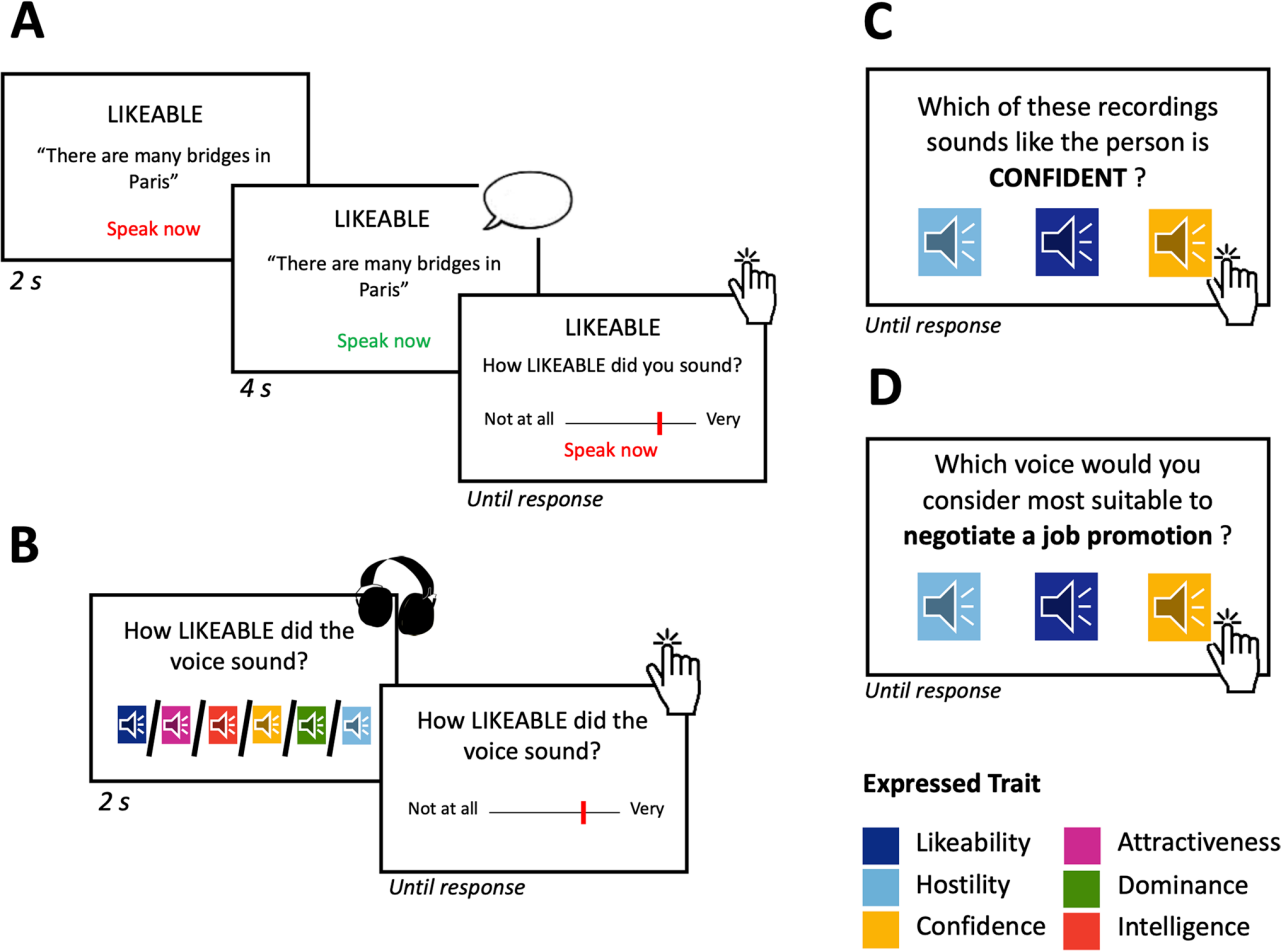
Example trial structure for the paradigms used in Experiments 1–3. (**A**) Example trial for the voice recordings (Experiment 1). (**B**) Example trial structure for the rating task, in which listeners rated how well they thought a voice expressed a specific trait (Experiment 1). (**C**) Example trial for the three-way forced-choice paradigm for Experiment 2, in which listeners picked the voice that they thought best expressed a specific trait out of three voice options. (**D**) Example trial for the three-way force-choice paradigm in Experiment 3, in which listeners picked the voice that they thought was most suitable for a real-life scenario

Talkers recorded sentences first in their neutral voice, followed by recordings where talkers modulated their voice along the six social traits. Within each recording trial participants saw the target trait (‘neutral’ in the first part, or a given social trait in the social vocal modulation task) and the exemplar sentence on the screen, together with a cue informing them when to speak (see Fig. 1A). At the beginning of each recording trial, participants had 2 s to prepare, after which the speech cue changed its color to green, marking the start of the recording. After 4 s, the speech cue turned back to red, indicating the end of the recording. Participants were then presented with a visual analogue scale to rate how much they thought they sounded like the target trait in the preceding recording trial (see Fig. 1B). The response scale ranged from ‘not at all [trait]’ (= 0) to ‘very [trait]’ (= 100). Each social trait × sentence combination was repeated in four subsequent trials. Thus, talkers produced 72 sentences (6 social traits × 3 sentences × 4 repetitions), which took approximately 30 min to complete. Visual cues were presented using the Psychophysics toolbox (Brainard, 1997; Kleiner et al., 2007) in MATLAB (version 2016a, the Mathworks, Natick, MA, USA). Recordings were obtained on a RØDE NT1-A 1 cardioid condenser microphone (Silverwater, Sydney, NSW, Australia) in an anechoic-chamber.

Based on these performance ratings obtained after each recording, we selected one representative recording per participant for each trait condition that had received the participant’s own maximal intensity rating for that trait and neutral voice recording. In cases where multiple recordings had the same maximal rating, we selected one recording at random.

#### Listener ratings of the recordings

Forty naïve listeners (age *M* = 25.38 years, *SD* = 5.20 years; nine male; 39 native German talkers, one Polish with German C2 level) were presented with the selected voice recordings obtained in the vocal modulation task. The sample size was based on optimal counter-balancing of talker recording to each listener. Naïve listeners were recruited via the subject databases of University of Mannheim and the Central Institute of Mental Health. Raters received a monetary reward (10 € per hour) or an equivalent in student credit points for their participation and gave their full informed written consent prior to participation.

Each listener heard a subset of ten talkers, where each talker was heard and rated by at least ten different raters (Guldner et al., 2020; Hughes et al., 2014). For each talker, one recording of each social trait and one recording of their neutral voice was included in the task. These recordings were presented in randomized order to listeners in six rating blocks, where each block was dedicated to rating how much a given voice expressed one of the social target traits, thus in each trial each listener rated each recording on a single trait to avoid spill-over effects (see Fig. 1B). Block order was also randomized. Listeners rated each recording on all traits on 7-point Likert scales measuring the intensity of expression of the rated trait in the voice from not at all (= 1) to very much (= 7). Stimuli were presented using the Psychophysics Toolbox (Brainard, 1997; Kleiner et al., 2007) in MATLAB (version 2016a, the Mathwork, Natick, MA, USA) on headphones (Sennheiser, Wedemark-Wennebostel, Germany) in an anechoic chamber.

### Data analyses

Data analyses were conducted in *R* (http://www.R-project.org/). To assess the effect of social vocal modulation, we subtracted the mean trait ratings for the neutral voice recordings on a given trait scale from the mean ratings obtained on this trait scale for each modulated voice sample, per talker. To illustrate this with an example: for intelligence, we separately subtracted the mean ‘intelligent’ ratings for the neutral voice from the mean ‘intelligent’ ratings for the attractive, likeable, hostile, intelligent, confident and dominant voice. Thus, we obtained the change in mean ratings as a function of each expressed trait, on each rated trait (henceforth referred to as ∆-ratings). The ∆-ratings were then analyzed in the framework of linear mixed-effects models (*lme4* package in *R;* Bates et al., 2015), separately for each rated trait. We implemented a priori defined planned treatment contrasts comparing the congruent social trait condition (when the trait expression and trait rating coincided, e.g., ∆-ratings of intelligence for intelligence modulations) to ratings of all other social conditions (when the trait expression and the trait rating did not coincide, e.g., ∆-ratings of intelligence for likeability modulations). Thus, we tested directly whether sounds from the congruent trait condition received significantly higher trait ratings on that trait than all other voice modulation conditions. Each model included mean naïve ratings from one of the trait conditions as the outcome variable, the expressed trait as a fixed effect term, talker as a random intercept, and talker sex as a nuisance variable (OSM S4 shows that there were no effects of talker sex). Likelihood ratio tests were performed to test the effect of trait expression on the ∆-ratings by comparing the models with fixed effects to the null models with only the random intercepts.

Next, we entered all z-transformed ratings for the neutral voice recordings and all z-transformed ∆-ratings from all modulated voice recordings into separate principal component analyses (PCA). In order to interpret the PCA components, we computed univariate one-way ANOVAs testing the effect of the social vocal control condition on each PCA component. Planned sum contrasts were then computed to test whether the recordings that were obtained from a particular social vocal control condition were significantly different from the overall mean. All principal component analyses were conducted using the package *FactoMineR* in *R* (Lê et al., 2008).

## Results

### Efficacy in social vocal control

**Fig. 2:**
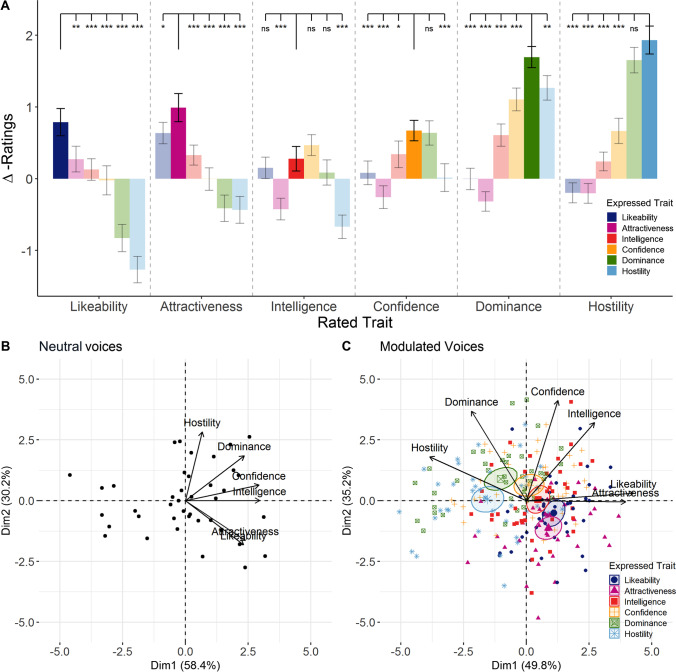
Naïve listener ratings of vocal modulations from Experiment 1*.* (**A**) Mean changes in trait ratings from neutral voices (∆-ratings) evoked by social voice modulations. Planned contrasts show differences in trait ratings over all social vocal control conditions. (**B**) Biplot of principal components for neutral voice recordings. (**C**) Biplot of principal components for ∆-ratings of modulated voice recordings. *** *p* < .001, ** *p* < .01, * *p* < .05. Error bars are standard errors. Ellipses represent 95% confidence intervals around the group means of expressed traits

Inter-rater reliability (Cronbach’s *α*) for the modulated voice ratings was .86 for all traits (95% confidence interval (CI): .85 - .87; within trait categories all Cronbach’s *α*s ≥ .83, see OSM Table S2). There were significant changes in mean intensity trait ratings evoked by social vocal modulation compared to neutral voices (∆-ratings) for all expressed traits, all *χ*^2^s(5) > 45.11, all *p*s < .001 (see Fig. 2A; descriptive results from naïve ratings on neutral voices are reported in OSM Fig. S3). To assess the specificity of social vocal modulation for each trait, we computed planned contrasts to test whether congruent trait ∆-ratings (when the expressed trait and the rated trait coincided) were rated significantly higher than incongruent traits (when expressed traits did not coincide with trait ratings).

Likeable, attractive and hostile voice modulations evoked congruent trait ratings that were significantly higher than all incongruent trait ratings, evidencing the specificity of the voice modulation. Intelligent and confident voice modulations showed less distinct evoked rating profiles, with some incongruent trait ratings being not significantly different from the congruent traits. Confident, dominant and likeable voice modulations led to increased ratings of intelligence, whereas dominant and confident voice modulations evoked higher confidence ratings. Lastly, dominant voice modulations were also perceived to be more hostile. Figure 2 shows the results from linear mixed effect models for each modulated trait (detailed comparisons are reported in OSM S4).

#### Social voice space for modulated voices

Inter-rater reliability for neutral voice recordings was comparable to previous work (Cronbach’s *α* = .85, see OSM Table S2; McAleer et al., 2014). We entered all trait ratings obtained for the neutral voice recordings into a principal component analysis to explore the organization of perceived vocal information along the dimensions of the social voice space (McAleer et al., 2014). The data were adequate for principal component analysis with appropriate inter-correlations (KMO = .73, with individual KMO > .53; Bartlett’s test of sphericity, *χ*^2^(15) = 209.14, *p* < .001). The first two components cumulatively explained 88.7% of the total variance (PC1 58.41%, PC2 30.25%, PC3 3.1%; see Fig. 2B and Table 1). Confidence and intelligence ratings loaded positively and most strongly on the first principal component. For the second component, hostility loaded positively, whereas likeability and attractiveness loaded negatively.

**Table 1:** Principal component loadings of all social traits and explained variance

PCA on …	Ratings of neutral voices		∆-Ratings of modulated voices	
Trait	PC1	PC2	PC1	PC2
Likeability	.74	-.58	.**93**	.07
Attractiveness	.76	-.52	**.86**	-.01
Intelligence	**.94**	.00	.59	.70
Confidence	**.94**	.21	.28	**.90**
Dominance	.75	.59	-.48	**.81**
Hostility	.21	**.90**	**-.84**	.40
Explained Variance (%)	58.41	30.25	49.82	35.23

Loadings represent the correlations of the trait judgments with the first two principal components as calculated including all six social traits. Correlations above .8 are highlighted in bold

We next examined the principal components of the changes in trait ratings induced by the modulated voice recordings. We entered the ∆-ratings of all recordings of trait modulations into a PCA. The sample contained sufficiently large inter-correlation between items (KMO = .72, with individual KMO > .65; Bartlett’s test of sphericity, *χ*^*2*^(15) = 1049.96, *p* < .001). The first two components cumulatively explained 85.1% of the total variance (PC1 49.8%, PC2 35.2%, PC3 6.1%; see Fig. 2C and Table 1). Likeability and attractiveness ratings loaded highly positively on the first component, while hostility ratings showed highly negative loadings. Dominance and confidence ratings loaded most strongly on the second component (see also OSM S5 for PCA of modulated voice based on non-normalized ratings).

The trait expressed in the voice had a significant effect on PC1, *R*^2^ = .31, *p* < .001. The traits likeability, *b* = 1.1,* p* < .001, and attractiveness, *b* = 0.88,* p* < .001, associated positively, while hostility, *b* = -1.54,* p* < .001, and dominance, *b* = -1.02,* p* < .001, associated negatively with PC1. For PC2, there was also a significant effect of expressed trait on the component with *R*^2^ = .22, *p* < .001. Here, dominance, *b* = 0.9,* p* < .001, and confidence, *b* = .63,* p* < .001, were positively associated with PC2, while likeability, *b* = -.51,* p* < .001, and attractiveness, *b* = -1.12,* p* < .001, were negatively associated.

## Discussion

In this experiment, we have shown that talkers can indeed volitionally modulate their voice to affect listeners’ impressions in a specific manner for likeability, attractiveness, dominance and hostility traits. More mixed results were found for expressed intelligence and confidence, where intended traits were at times perceptually associated with other traits: while expressions of likeability, attractiveness, dominance and hostility evoked specific trait impressions (e.g., likeable modulations were more likeable than any other modulation), confident and intelligent voice modulations appeared to be less specific (e.g., intelligent modulations evoked comparable intelligence *and* confidence ratings). A possible explanation might be that intelligent and confident voice modulations were more difficult to achieve by talkers (Guldner et al., 2020). However, we note that talkers were similarly satisfied with all their trait expressions (see OSM S6), and the listeners in our perceptual tasks tended to agree in their perception of these expressed traits with inter-rater agreement similar to previous studies on unmodulated voices (Mahrholz et al., 2018; McAleer et al., 2014). An alternative explanation is that cues to confidence and intelligence may be functionally similar in the voice, and more specifically differentiable only in the presence of additional information such as context, speech content, posture (Satchell et al., 2017) or facial appearance/expressions (Willis & Todorov, 2006), which might influence the weighting of social information for person judgement (Rezlescu et al., 2015). Moreover, the discriminability of traits along the opposite poles of the affiliation dimension (likeability and hostility) might be higher than traits that cluster within more similar locations in the trait space (e.g., intelligence and confidence). In future work, it might be useful to explore to model traits along both poles of the competence dimension more explicitly (e.g., including expressions of ‘submission’ as well as dominance).

In our PCA analysis, we replicated the finding of a common underlying low-dimensional trait space for neutral voices, with dimensions allied to affiliation and competence (McAleer et al., 2014). However, our analysis of modulated voices provides novel evidence that trait ratings evoked by intentional voice modulations can also be described by these same two dimensions. This finding suggests that talkers can specifically amplify the perception of social traits within the same shared trait space, in goal-directed ways.

From these initial findings, we therefore suggest that intentional voice modulation can be exploited by talkers to navigate social space, such as in social scenarios where a certain impression is targeted (e.g., to convey competence or attractiveness; Feinberg et al., 2005; Pavela Banai et al., 2017; Schroeder & Epley, 2015; Tigue et al., 2012).

## Experiment 2

### Introduction

Experiment 1 found that intentional voice modulations affect listeners trait impressions in a specific manner via dimensional trait ratings of individual sentences. In Experiment 2, we tested the replicability and generalisability of the findings of Experiment 1, by using a task in which listeners were asked to select the most suitable match to a trait label from three presented recordings. Thus, participants were required to trade off different recordings against each other for suitability.

### Methods

#### Participants

Forty naïve listeners (age *M* = 29.11 years, *SD* = 5.38 years; 20 males, one not disclosed) were recruited from a sample of native German talkers via Prolific.co. All participants were aged 18–40 years, and had no language-related disorders, literacy difficulties or hearing difficulties. Participants were paid £2.75 for participation, based on an hourly rate of £7.50. The study was approved by the University College London Ethics Committee. All participants read an information sheet and provided informed consent prior to data collection.

#### Stimuli

Audio stimuli consisted of voice recordings from the social vocal control speech production task in Experiment 1. For each talker, we selected the highest self-rated modulated speech token for the social trait labels of hostility, likeability and confidence. These three traits were focused upon as they were clearly distinct from each other in the two-dimensional social voice space demonstrated by the principal component analysis in the previous study. They furthermore showed different levels of specificity and intensity of perceived modulation in Experiment 1. For example, likeability was distinct from all other traits, while hostility and confidence were not distinct from dominance. Similarly, mean ∆-ratings are substantially higher for hostile voices than for likeable and confident voices.

#### Procedure

The study was administered online, programmed and hosted on the Gorilla Experiment Platform (www.gorilla.sc; Anwyl-Irvine et al., 2020). It comprised a three-alternative forced-choice task, in which participants selected one speech token from three exemplars to match a given social trait label. The task consisted of three blocks, which specified whether participants should select the exemplar that sounded the most hostile, likeable or confident. The order of blocks was counterbalanced between participants. At the beginning of each block, participants were instructed on the target social trait to detect, along with a relevant description (e.g., *Confidence: This person wants to give others the impression that they can rely on the person and their abilities*). The three social trait blocks were presented in a randomised order, and each consisted of 44 trials. During 90% of trials, the array of three speech exemplars always included one example from each social trait modulation (hostile, likeable and confident) uttered by the same talker, with play buttons presented on-screen in a counterbalanced left-to-right order. Participants heard each of the three exemplars in succession (left-to-right), before having the option to replay any of the recordings as many times as they wished. Finally, participants indicated their final choice for which exemplar matched the specific social trait for the given block. The participants completed this decision for 40 different talkers (one trial per talker; see Fig. 1C). The remaining 10% of trials served as a vigilance check, in which all three speech recordings provided an identical verbal instruction (e.g., *“Select the second option”*). Performance on vigilance trials was used to check attention and task compliance. Participants also completed a headphone check before completing the experiment, in order to ensure that they could hear the audio stimuli.

### Data analysis

Voice modulation effectiveness was measured based on the proportion of instances when the selected trait (the voice modulation exemplar chosen by the listener for a given social trait label) aligned with the intended expressed trait (the voice modulation exemplar in which the talker was attempting to convey the corresponding social trait label). Listeners’ responses were visualised and analysed using the *ggplot2* (Wickham, 2016), *caret* (Kuhn, 2008) and *mlogit* (Croissant, 2020) packages in *R*. First, we conducted a confusion analysis, which determined how often listeners perceived voice modulation exemplars as characteristic of the social trait intended, as well how often other intended expressed social traits were misclassified. Next, we ran a multinomial logistic regression model to statistically infer performance in individual conditions, and to incorporate variance from random effects of Talker ID and Listener ID. Beta (β) and odds ratios (ORs) are used to report effect sizes. β is the logit transformed fixed effect coefficient, which refers to the estimated difference between different voice modulations being selected having controlled for random effects. ORs (derived from β) measure the difference in odds of a voice modulation expressing a specific social trait being selected over a voice modulation expressing an alternative social trait.

## Results

**Fig. 3:**
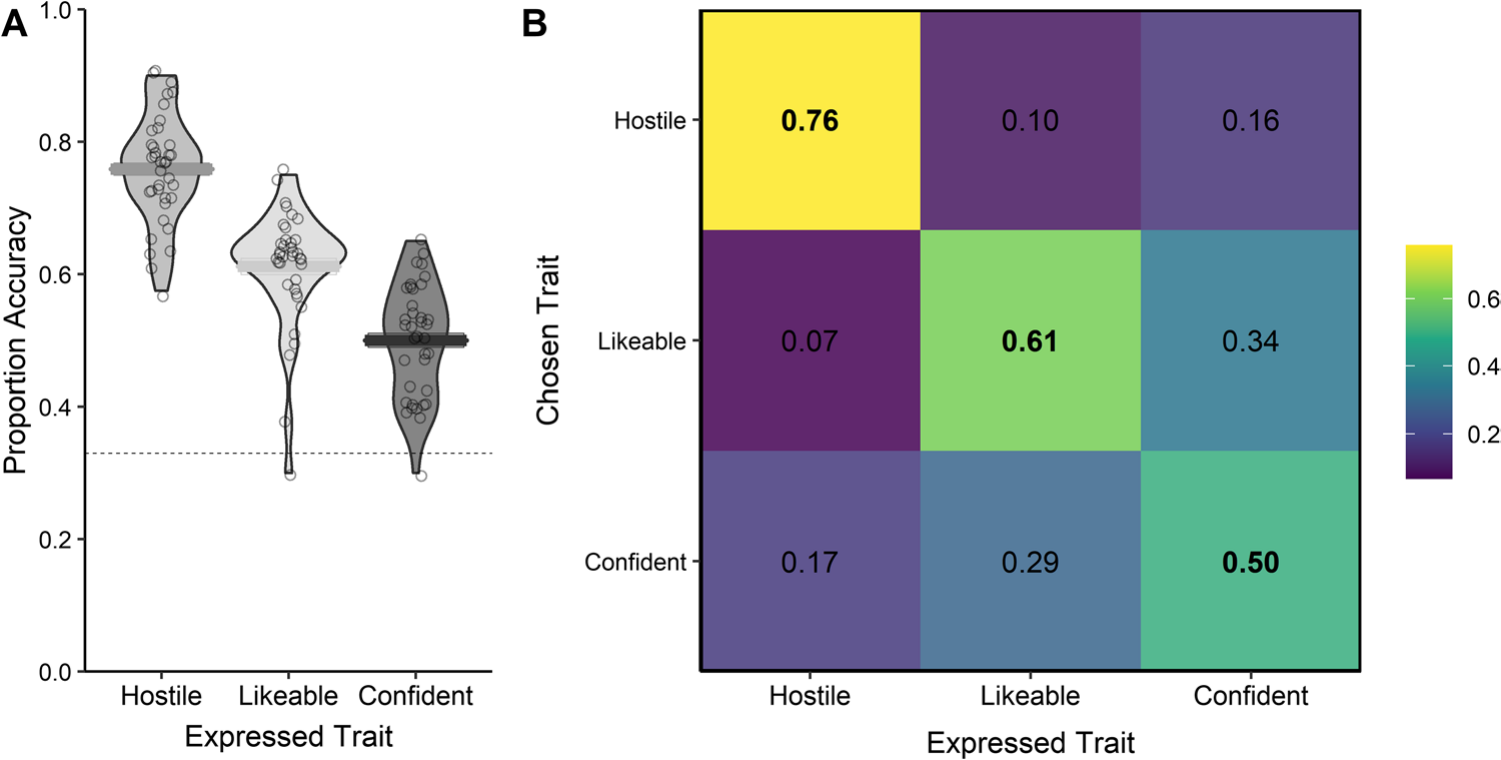
Proportion of responses for each trait modulation and corresponding accuracy for Experiment 2. (**A**) Violin plot showing the accuracy of response for each listener by modulated trait. Horizontal bars show the mean accuracy for the relevant modulated trait category across listeners. The dashed lines represent chance performance. (**B**) Confusion matrix showing the proportion of each trait selected (chosen trait) to match each modulated trait condition (expressed trait) across listeners and speakers

The confusion analysis indicated that naïve listeners demonstrated the sensitivity and specificity required to distinguish the voice modulation expressing the intended trait from other voice modulations by the same talker. The Kappa value (*K*) indicated moderately good performance, and the detection of the intended trait was significantly above the no information rate, or chance performance (*K* = 0.43, *p* < .001; Fig. 3). The multinomial logistic model indicated that naïve listeners were always significantly more likely to select the intended expressed trait relative to the other options. For instance, if a speaker modulated their voice to sound hostile, listeners were over 11 times more likely to consider them hostile compared to when they modulated their voice to sound likable (Table 2).

**Table 2:** Multinomial logistic model output for Experiment 2, indicating the likelihood of naïve listeners selecting the voice modulation expressing the intended social trait relative to voice modulations expressing alternative social traits

Intended trait	Alternative choice	OR	β	SE	z	*p*
Hostile	Confident	4.34	1.47	0.07	21.28	<0.001
	Likeable	11.46	2.44	0.11	23.15	<0.001
Likeable	Confident	2.09	0.74	0.06	12.65	<0.001
	Hostile	6.33	1.85	0.09	20.50	<0.001
Confident	Hostile	3.08	1.13	0.07	15.16	<0.001
	Likeable	1.48	0.39	0.06	6.77	<0.001

OR = odds ratio; SE= standard error

## Discussion

Experiment 2 conceptually replicates the findings from Experiment 1 using a substantially different paradigm. Specifically, the findings confirm that talkers’ intentional voice modulations can successfully convey social traits to naïve listeners, where listeners are able to selectively recognise expressed traits via categorical choices and in the presence of competitor stimuli. Intriguingly, as in Experiment 1, Experiment 2 also shows different levels of specificity across different traits, with hostile voices being the least confusable, followed by likeable and then confident voices. These differences in confusability may be driven by how these three traits are related to one another: Confidence falls between hostile and likeability on the low-dimensional voice space, thus potentially making it most confusable (Fig. 3B). Similarly, confusions also seem to reflect the magnitude of ∆-ratings among the three traits as observed in Experiment 1 (Fig. 3A). The observed differences in specificity for the different traits may thus be taken as further evidence that specific traits (e.g., confidence) do indeed map onto much broader concepts allied to affiliation and competence.

## Experiment 3

### Introduction

In Experiment 3, we investigated the functionality of vocal modulations, by asking whether intentional voice modulations can also be recognised and interpreted when listeners are provided with real-life scenarios (speaking in a job interview) rather than abstract trait labels (‘confident’).

### Methods

#### Participants

An additional 40 naïve listeners (age *M* = 28.53 years, *SD* = 5.41 years; 14 male) were recruited from a sample of native German talkers via Prolific.co. These participants met the same demographic criteria as those recruited for Experiment 2, although none of them participated in the previous study. Participants were paid £2.75 for participation, based on an hourly rate of £7.50. The study was approved by the University College London Ethics Committee. All participants read an information sheet and provided informed consent prior to data collection.

#### Stimuli

The audio stimuli were identical to the stimuli used in the three-alternative forced-choice task in Experiment 2. Stimuli were presented in the same arrays, following identical randomisation and counter-balancing procedures.

#### Procedure

The study was administered online, programmed and hosted on the Gorilla Experiment Platform (www.gorilla.sc; Anwyl-Irvine et al., 2020). The experiment again comprised a three-alternative forced-choice task, with a near-identical procedure to Experiment 2 (Fig. 1D). There was one key difference: participant judgements were based on suitability of a voice for a real-life social scenario, rather than alignment with a social trait description. These scenarios were based on situations in which one of the intended expressed social traits (hostility/likeability/confidence) would be advantageous in achieving a specific goal. The scenarios are outlined in Table 3.

**Table 3:** Scenario prompts presented to naïve listeners in Experiment 3

Social trait	Scenario
Hostile	Which voice would you consider most suitable to portray a villain in a film?
Likeable	Which voice would you consider most suitable to ask a new friend to go hiking with you?
Confident	Which voice would you consider most suitable to negotiate a job promotion?

We selected the scenarios via an online validation task in a separate sample of 40 native German participants. Participants were presented with 15 different scenarios and asked to decide how a talker should sound in the scenario presented. Participants chose one of three written descriptions of the social traits (hostile/likeable/confident). The three scenarios used in the experimental task were the scenarios most consistently associated with each of these social trait descriptions during the validation task.

### Data analysis

The data were analysed following the same procedure as Experiment 2. Scenario prompts were labelled using the social trait that they were associated with (e.g., voice most suitable to portray a villain in a film = hostile): voice modulation performance was then based on how often listeners selected the exemplar in which the talker aimed to express the social trait associated with the scenario prompt. We fitted a multinomial logistic regression incorporatingd nested random effects of Talker ID and Listener ID to estimate performance in individual conditions.

## Results

**Fig. 4:**
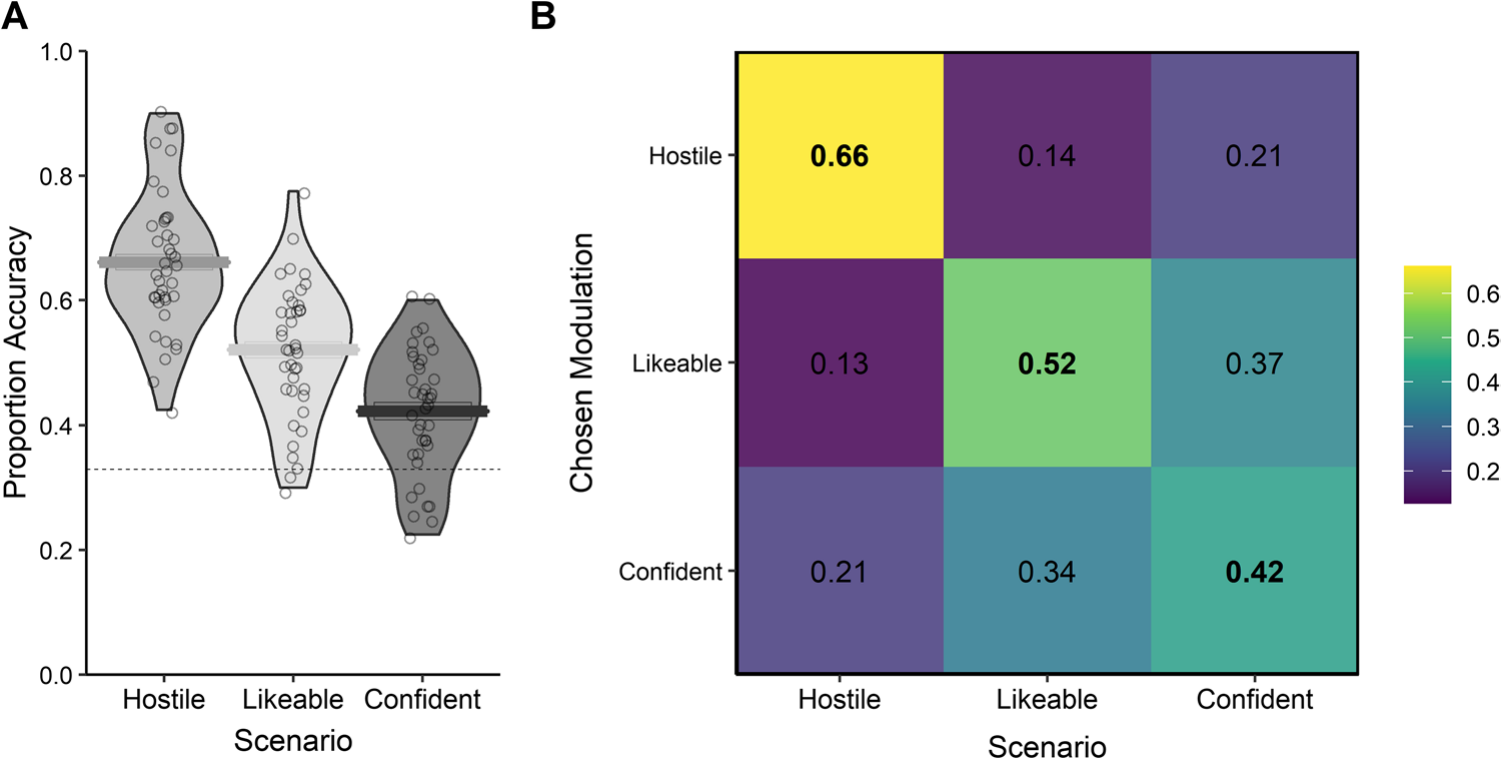
Proportion of responses for each trait modulation and corresponding accuracy for Experiment 3. (**A**) Violin plot showing the accuracy of response for each listener by modulated trait. Horizontal bars show the mean accuracy for the relevant modulated trait category across listeners. The dashed lines represent chance performance. (**B**) Confusion matrix showing the proportion of each trait selected (chosen modulation) to match each modulated trait scenario (scenario) across listeners and speakers

The confusion analysis showed listeners displayed fair performance at preferentially selecting the relevant voice modulation for a given scenario. Detection of the intended social trait modulation associated with the scenario was significantly above the no-information rate, or chance performance (*K* = 0.30, *p* < .001; Fig. 4). The multinomial logistic regression model confirmed that voice modulations intended to express the relevant social trait were preferentially selected over other voice modulations in all scenarios (Table 4). Descriptively speaking, the results of Experiment 3 closely resemble those of Experiment 2, with similar patterns of confusability arising across the three traits. Here, if a speaker modulated their voice to sound hostile, listeners were over five times more likely to consider them suitable to portray a villain compared to when they modulated their voice to sound likeable. Simultaneously, if the speaker modulated their voice to sound likeable, listeners were more than three times more likely to find their voice suitable to socialize with others than if the speaker intended to sound hostile (Table 4).

**Table 4:** Multinomial logistic model output for Experiment 3, indicating the likelihood of naïve listeners selecting the voice modulation expressing the intended social trait related to a scenario, relative to voice modulations expressing alternative and less relevant social traits

Scenario-related intended trait	Alternative choice	OR	β	SE	z	*p*
Hostile	Confident	3.12	1.14	0.06	18.24	<0.001
	Likeable	5.21	1.65	0.08	21.55	<0.001
Likeable	Confident	1.53	0.42	0.06	7.70	<0.001
	Hostile	3.75	1.32	0.08	17.51	<0.001
Confident	Hostile	2.02	0.70	0.07	10.53	<0.001
	Likeable	1.15	0.14	0.06	2.50	<0.05

OR = odds ratio; SE = standard error

## Discussion

Experiment 3 shows that, even without being given specific trait labels, listeners are able to recognise the function of the social traits expressed in volitionally modulated voices. Specifically, we show that the changes in trait perception that talkers can evoke in listeners can be generalised to real-life scenarios, thus showing that while specific (to varying degrees), intentional voice modulations can be functional in nature and are not restricted to abstract classification based on trait labels.

### General discussion

Talkers can change their voices strategically in interactions to convey meaningful non-verbal information that helps them to achieve social goals. Our study comprehensively quantifies the perceptual effects of such intentional voice changes, showing that the voice can be a highly effective vehicle to change a listener’s impression of a talker. We show that, through intentional voice modulation, talkers were able to specifically amplify listeners’ percepts of most target traits, effectively traversing social trait space in goal-directed ways (see also Hughes et al., 2014). This work is novel as most research to date has focussed on defining the dimensions of social trait space in different modalities (e.g., voices and faces; McAleer et al., 2014; or social groups; Fiske et al., 2007), and any previous works aiming to manipulate perceptual changes along the social space dimensions for voices have done so using artificial manipulations of voice recordings, such as changes to voice pitch (Belin et al., 2017), rather than intentional modulations produced by real-life talkers.

In our set of experiments, we find that the effect of social voice modulations can be measured across different types of experimental designs (ratings vs. forced-choice) and contexts (e.g., abstract labels vs real-life social situations). We highlight in particular that intentional voice modulations were effective in social settings (Experiment 3), such that listeners tended to choose a matching voice modulation to a social goal without any explicit information about the voice or the talker. Instead, selection was driven by the voice perceived to be beneficial for achieving a social goal. This indicates that intentional voice modulations not only influence listeners’ perception of social traits attributed to talkers, they also implicitly influence listeners’ decision making, which can be beneficial for achieving meaningful social goals. Additionally, there was a substantial overlap between listener judgements of traits and the subsequently selected scenarios: those recordings assigned, for example, a confident label by listeners in Experiment 2, were also chosen for the corresponding social scenario in Experiment 3 (see OSM S7). This highlights the efficacy of voice modulations from talkers in evoking robust and consistent listener impressions of intended traits across multiple decision contexts.

But how might these results translate to real-life interactions? In naturalistic human communication, individuals play the role of both the sender and the receiver of social cues, and exchanges unfold over time with contextual information being available to further guide interactions. In our study, talkers and listeners, however, acted in isolation and on the basis of single sentences. On the one hand, this might have supported the perceptual discrimination of some traits. In the absence of an interlocutor, as in our study, talkers most likely relied on more typical, easy-to-recognise expressions of social traits, adhering to cultural display rules learned through observational learning of reinforcement contingencies (shown previously in studies of child talkers; Cartei et al., 2014, 2020).

On the other hand, the absence of the interlocutor and a single sample for each talker might have impeded the efficacy of voice modulations. Confident voice modulations, for instance, evoked less specific rating profiles. This is not in itself surprising, given that nonverbal social signals are often ambiguous (DePaulo, 1992; Hellbernd & Sammler, 2018), but it suggests that additional contextual information might be important to interpret and express some traits with specificity. This type of information might be gathered over time: the interlocutor can retrieve information about intentions, affective state or feelings from how an interaction unfolds (e.g., Gregory et al., 1993; Manson et al., 2013; Pardo et al., 2012). With this added information, the expression and interpretation of vocal modulations between talker and listener might become more fine-tuned to each other over the time course of the exchange, leading to more efficient communication.

We also note that in the current study, listeners trait impressions were based solely on talkers’ voices, while real-life interactions offer additional information about a person’s traits to be gleaned from multimodal and contextual information (e.g., Rezlescu et al., 2015). For example, in a job interview functional use of a confident vocal modulation might be supported by a smart attire, posture or other status symbols. Lastly, and as touched upon above, talker or listener attributes might influence the efficacy of voice modulations, such as their vocal control ability, differences in extra- or intra-psychological aspects (e.g., enculturation, training or empathy, respectively). While the basic findings of this study should replicate, we speculate that in real-life situations, talkers and listeners might be even more effective in expressing and decoding particular social traits based on being able to pull in additional sources of information.

Large-scale future studies using conversational interactions would be useful in understanding dynamic vocal modulation in more naturalistic social settings. To date studies using naturalistic settings mainly consider a single context, often dating scenarios/mate selection (e.g., McFarland et al., 2013; Ranganath et al., 2009). In these studies, participants do not intentionally modulate their voice, but spontaneous (vocal) behaviour as measured as a function of the social scenario (such as evoking a desirable impression on the interlocutor; e.g., Pisanski et al., 2018). Given our findings that *intentional* vocal behaviour might rely on a common perceptual social code structured by a multi-dimensions voice space, there is a need to further explore how talkers skilfully traverse this space to achieve a wide range of social goals, going far beyond contexts of mate selection.

We acknowledge that the current study’s focus was on the perceptual efficacy and function of vocal modulations rather than their acoustic bases – however, with a larger sample size it would be of interest to investigate whether specific trait impressions are conveyed via *specific* acoustic cues linked to each trait or rather through continuous dimensional changes that map onto the axes of social voice space (see OSM S8 for acoustic parameters). Future work collecting data on the perceptibility of modulations, for example via authenticity judgements (“How *genuinely* confident does this person sound?”) may yield further insights into the limits within which such acoustic changes are effective.

In summary, we show that listeners can volitionally control their voices to express social traits, which can in turn be successfully perceived by listeners. Being able to amplify social traits through the voice can have important advantages: it can signal affiliation (e.g., Giles et al., 2016), support clear communication of a talker’s own internal state, and be functional to a social goal, insofar as perception along the social trait space dimensions can bias subsequent social behaviour. This has been shown, for instance, for political votes (Pavela Banai et al., 2017; Tigue et al., 2012), job interviews (Schroeder & Epley, 2015) or trust scenarios (Montano et al., 2017). Here we find support for this notion, showing that vocal trait expressions were assigned to concordant social goals in scenario vignettes (e.g., using a confident voice to negotiate a promotion), thereby underlining intentional vocal behaviour can be a relevant means in managing how we are perceived by others.

### Supplementary Information

Below is the link to the electronic supplementary material.

Supplementary file1 (DOC 415 KB)

## Data Availability

The data for Experiments 1, 2 and 3 are publicly accessible https://osf.io/avkby/?view_only=cd24e889c98448348027f4907a45f09c. The analysis code and materials used in the reported studies are publicly accessible upon reasonable request to the authors.
