# Supplementary material for: Human talkers change their voices to elicit specific trait percepts
Source: Psychon Bull Rev. 2023 Jul 28;31(1):209–22. doi: 10.3758/s13423-023-02333-y (PMC10866754; doi:10.3758/s13423-023-02333-y)
Supplement: Supplementary file 1 — Supplementary file1 (DOC 415 KB) [file 13423_2023_2333_MOESM1_ESM.doc]

**Supplementary Materials**

**S1. Vocal Modulation instructions.**

Attractive: Speak as if you were trying to impress someone in whom you are romantically interested.

Dominant: Speak as if you were trying to assert authority.

Intelligent: Speak as if they were at a scholarly conference giving a presentation.

Confident: Speak as if you were trying to make others trust and believe in your ability to do something.

Likeable: Speak as if you were trying to be liked by someone else.

Hostile: Speak as if you want to distance yourself from someone else.

**S2. Inter-rater reliability.** Cronbach’s α for neutral voice recordings and expressed traits. Table S2 shows Cronbach’s α scores grouped by each expressed trait and the neutral voice ratings among the 10 raters of each recording.

| **Trait** | **Cronbach’s α** | **95% CI (lower - upper)** |
| --- | --- | --- |
| Likeability | .87 | .84 - .89 |
| Attractiveness | .86 | .84 - .89 |
| Intelligence | .83 | .8 - .87 |
| Confidence | .85 | .82 - .88 |
| Dominance | .86 | .84 - .89 |
| Hostility | .86 | .84 - .89 |
| Neutral | .85 | .83 - .85 |
| All recordings | .86 | .85 - .87 |

**Figure S3. Mean ratings of neutral voices.** Naïve listener ratings obtained for the neutral voice recordings on all traits for each speaker given on a 7-point Likert-scale ranging from 1 (=not at all) to 7 (very). Mean_Attractiveness_=3.01, SD=0.99; Mean_Dominance_=2.88, SD=0.96; Mean_Hostility_=2.19, SD=0.76; Mean_Intelligence_=3.85, SD=1.10; Mean_Confidence_=3.86, SD=1.20; Mean_Likeability_=3.73, SD=1.03.

**
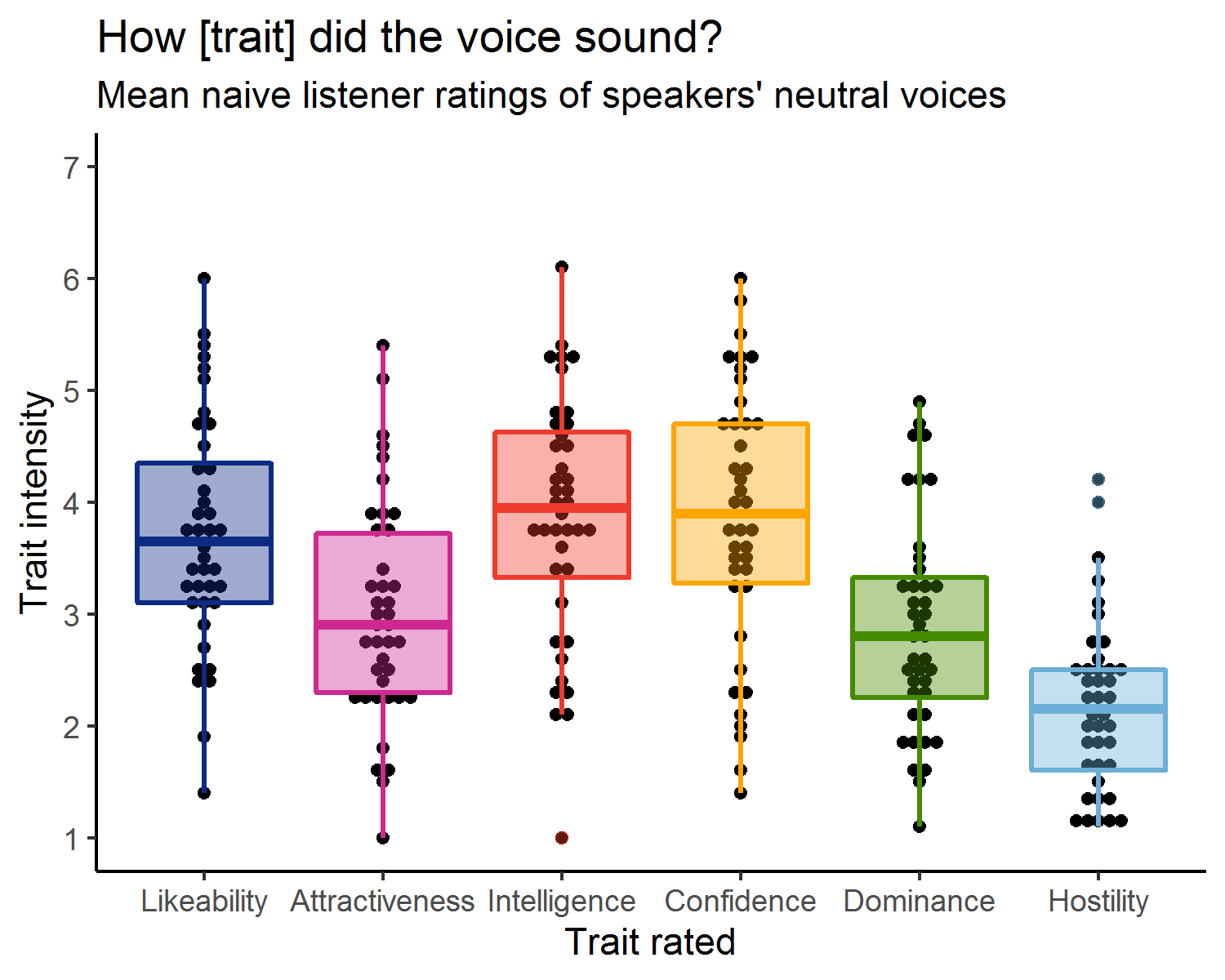
**

**S4. Outcomes from linear mixed effects models for trait comparisons on ∆-ratings.** Likeable voice modulations were rated as significantly more likeable than neutral voices ∆-rating of .87, *t*(84.35)=4.28, *p*<.001, and were also rated as sounding more likeable than any other trait modulation (all *b*s< -.51, all *t*s(200)> -2.9, all *p*s≤ .01). Talker sex did not influence listener ratings of likeability (*b*=-.26, *t*(40)=-.88, *p*=.38).

Attractive voice modulations were rated as sounding significantly more attractive than neutral voices (mean ∆-rating =.99, *t*(89.78)=5.26, *p*<.001), and any other trait modulation (all *b*s< -.36, all *t*s(200)> -2.04, all *p*s≤ .042). There was no effect of talker sex on attractiveness ratings (*b*=-.02, *t*(40)=-.06, *p*=.95).

Intelligent voice modulations did not evoke higher ratings in intelligence compared to neutral voices (mean ∆-rating=.25, *t*(76.50)=1.39, *p*=.17) and were perceived as sounding similarly intelligent to confident (*b*=.19, *t*(200)=1.27, *p*=.21), dominant (*b*=-.19, *t*(200)=-1.29, *p*=.20), and likeable voice modulations (*b*=-.13, *t*(200)=-.85, *p*=.40). However, hostile (*b*=-.95, *t*(200)=-6.34, *p*<.001) and attractive (*b*=-.70, *t*(200)=-4.70, *p*<.001) voice modulations, were rated as sounding significantly less intelligent compared to intelligent voice modulations. There was no effect of talker sex on intelligence ratings (*b*=.08, *t*(40)=-.31, *p*=.76).

Compared to neutral voice samples, confident voice modulations evoked significantly higher ratings on confidence (mean ∆-rating=.61, *t*(80.41)=3.22, *p*<.01). Apart from dominant voice modulations, which did not significantly differ from confident voice modulations on perceived confidence (*b*=-.03, *t*(200)=-.20, *p*=.84), all other trait modulations were rated as significantly less confident (all *b*s< -.33, all *t*s(200)> -2.02, all *p*s≤ .044). Female and male talkers were rated as similarly confident across vocal modulations (*b*=.18, *t*(40)=-.64, *p*=.52).

Dominant vocal modulations were perceived as significantly more dominant than neutral voices (mean ∆-rating=1.65, *t*(92.28)=9.71, *p*<.001), and all other trait modulations (all *b*s< -.43, all *t*s(200)> -2.72, all *p*s≤ .007), independent of talker sex (*b*=.15, *t*(40)=.65, *p*=.52).

Lastly, hostile voice modulations sounded more hostile than neutral voices ∆-rating of 1.87, *t*(119.06)=10.72, *p*<.001, and were also perceived as more hostile than all other trait modulations (all *b*s< -1.27, all *t*s(200)> -6.93, all *p*s≤ .001) apart from dominant voice modulations, which were perceived as sounding similarly hostile (*b*=-.28, *t*(200)=-1.53, *p*=.13). Talker sex did not influence listener ratings of hostility (*b*=.19, *t*(40)=.85, *p*=.40).

**S5. Results of PCA of mean ratings on social traits for modulated voices.** The sample contained significantly large enough inter correlation between items to allow PCA analysis (KMO=.72, with individual KMO >.63; Bartlett’s test of sphericity, *χ2*(15) = 1345.10, *p*<.001). The first two components cumulatively explained 87% of the total variance (see S5 Table 1). **Figure S5** depicts a biplot of loadings on mean ratings of social traits on modulated voices grouped by the social traits expressing the voice including group centroids. Ellipses illustrate 95% confidence intervals on group centroids of social traits expressed in the voices. **Table S5** shows the loadings of social trait ratings on the two first principal components.

**
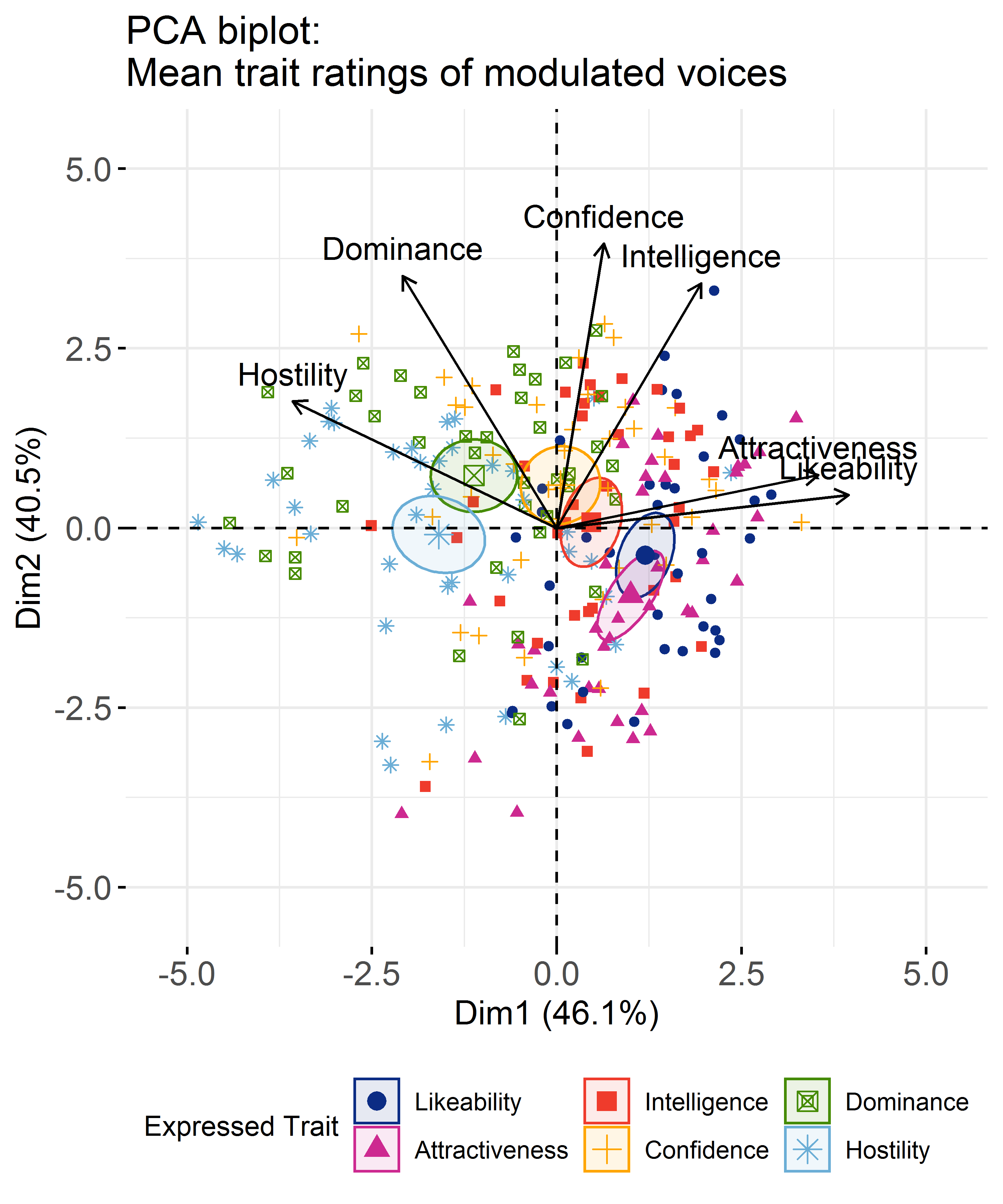
**

| Table S5. Principal component loadings of all social traits and explained variance on modulated voice ratings | | |
| --- | --- | --- |
| **Trait** | **PC1** | **PC2** |
| Likeability | .**9** | .3 |
| Attractiveness | .81 | .28 |
| Intelligence | .25 | **.91** |
| Confidence | .17 | **.95** |
| Dominance | -.66 | .71 |
| Hostility | **-.92** | .21 |
| Explained Variance (%) | 46.1 | 40.5 |
| *Note*. Loadings represent the correlations of the trait judgments with the first two principal components as calculated including all six social traits. Correlations above .9 are highlighted in bold. | | |

**Figure S6. Speakers’ mean performance ratings of voice modulations for all recordings (A) and for recordings selected for naïve ratings (B).** Speakers own performance ratings were obtained after each trail and were given on a visual analogue scale with anchors at 0 (=not at all) and 100 (=very). For each expressed trait and speaker, only those recordings that received maximal intensity ratings was passed on to naïve listeners for ratings.


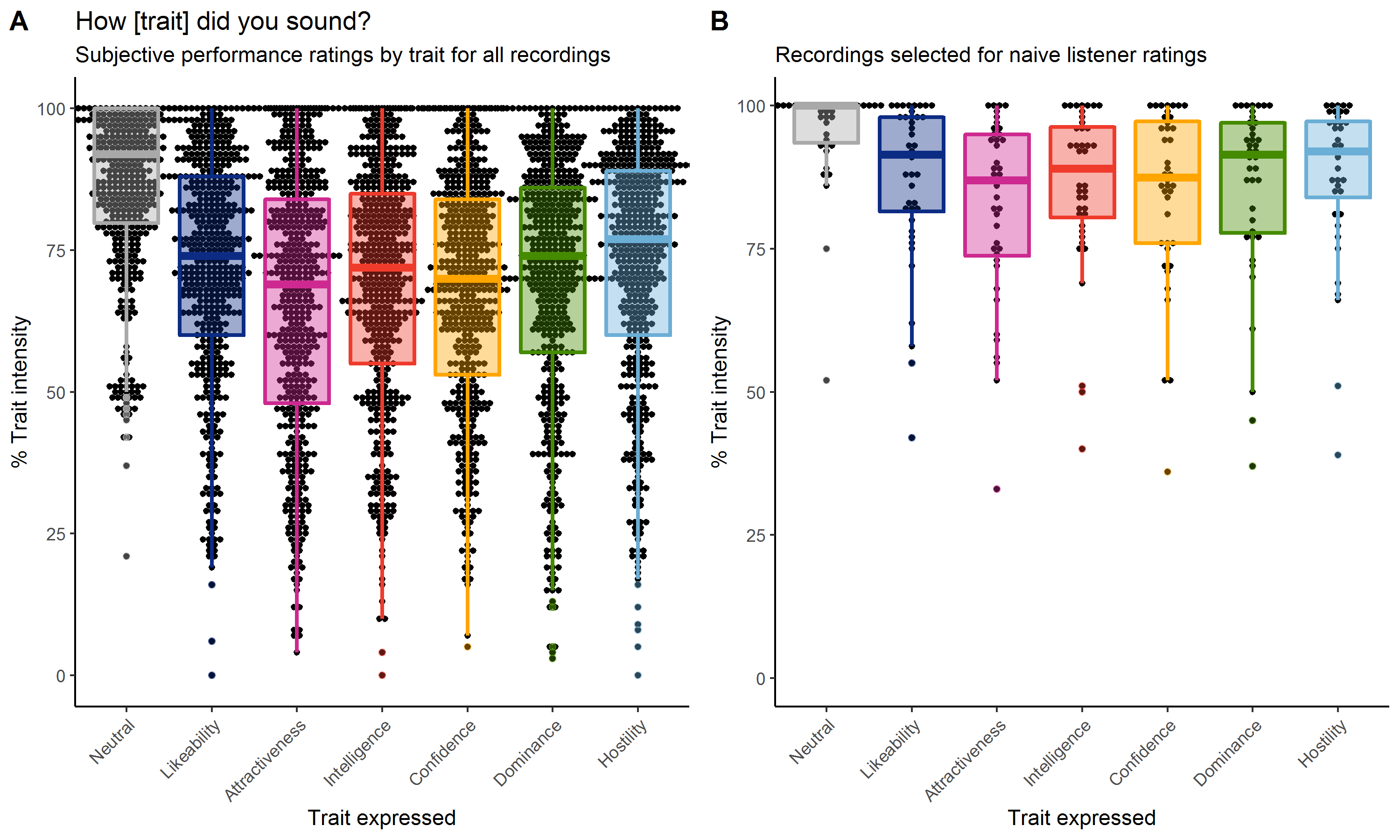


**A.** **All recordings.** Mean_Attractiveness_=65.1, SD=23.2; Mean_Dominance_=69.4, SD=22.1; Mean_Hostility_=72.2, SD=21.4; Mean_Intelligence_=68.3, SD=21.0; Mean_Confidence_=67.4, SD=20.7; Mean_Likeability_=71.6, SD=20.0; Mean_Neutral_=86.8, SD=15.2.

**B. Recordings selected for naïve ratings** Mean_Attractiveness_=82.1, SD=16.1; Mean_Dominance_=85.8, SD=15.6; Mean_Hostility_=87.8, SD=13.8; Mean_Intelligence_=85.9, SD=14.3; Mean_Confidence_=84.9, SD=15.2; Mean_Likeability_=87.1, SD=14.1; Mean_Neutral_=95.5, SD=8.96.

**S7. Overlap between voice modulations in Experiments 2 and 3.** We assigned each recording the trait that was most often assigned to it by listeners in Experiment 2. We then compared the assigned trait from Experiment 2 to the chosen trait scenario in Experiment 3. The confusion analysis showed a significant overlap between Experiments 2 and 3, with an accuracy of .82 (95% CI: 0.74-0.88), significantly different from chance (No-Information-Rate=.35, p<.0001, Kappa=0.72). McNemar’s test was not significant (*p*=0.96). Traits were assigned to the matching scenario with a sensitivity of .90 for hostile modulations, .83 for likeable, and .70 for confident modulations. Specificity for hostile voice modulations was .95, .88 for likeable and .89 for confident voice modulations (see Figure S7). This suggests that voices that were expressed, judged and chosen as a particular trait were also chosen to be functional in a matching social scenario.


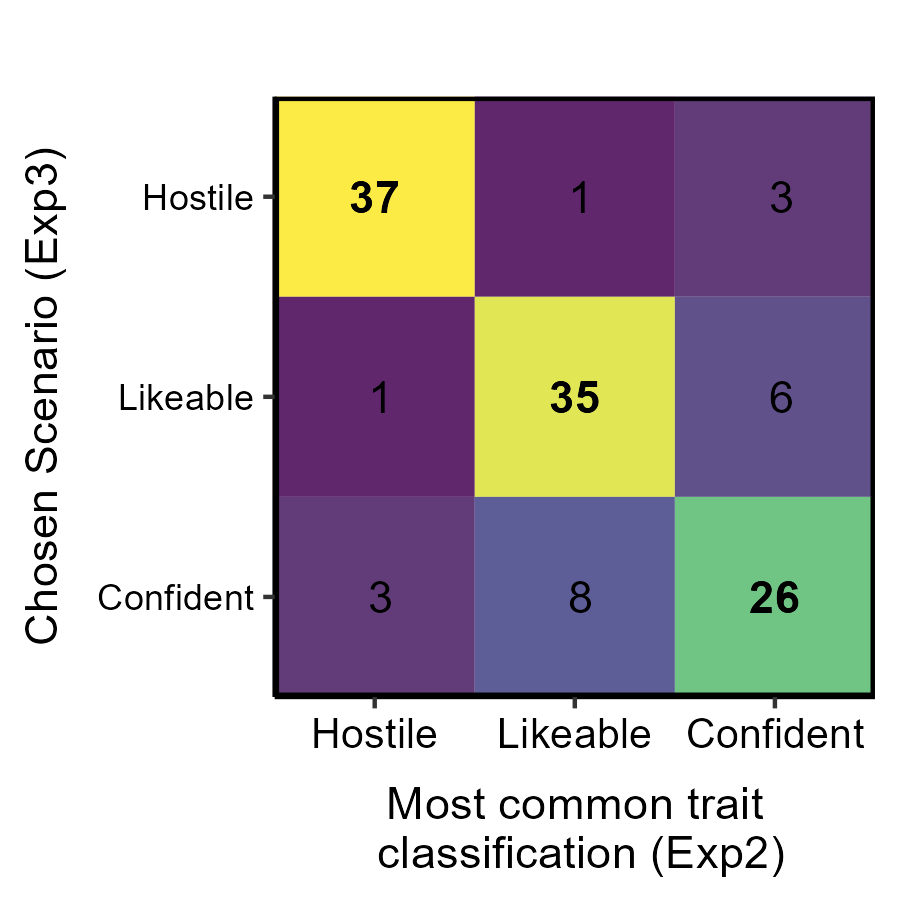


**Figure S7.** Confusion matrix showing total number of recordings with assigned trait membership from experiment 2 and experiment 3. Because of the posteriori trait assignment based on experiment 2, the group sizes differ between traits.

| **Table S8.** Acoustic description of normal and modulated voices. | | | | | | | | |
| --- | --- | --- | --- | --- | --- | --- | --- | --- |
| **Expressed Trait** | **Duration (s)** | **Mean F0**  **(Hz)** | **SD F0**  **(Hz)** | **Mean Intensity (dB)** | **% Unvoiced** | **HNR** | **Spectral centre of gravity (Hz)** | **SD spectrum (Hz)** |
| Normal | 1.75 ± .32 | 205.96 ± 52.45 | 84.71 ± 43.28 | 77.82 ± 3.28 | 28.59 ± 11.41 | 14.20 ± 3.33 | 527.99 ± 290.46 | 1178.46 ± 766.4 |
| Likeable | 1.65 ± .29 | 213.61 ± 50.71 | 90.54 ± 30.37 | 78.66 ± 2.99 | 27.53 ± 10.8 | 14.38 ± 3.33 | 521.99 ± 260.54 | 1176.83 ± 743.6 |
| Attractive | 1.82 ± .28 | 194.41 ± 48.5 | 81.00 ± 33.32 | 76.85 ± 3.65 | 26.12 ± 10.93 | 14.47 ± 3.02 | 416.52 ± 242.89 | 934.14 ± 604.75 |
| Intelligent | 1.91 ± .39 | 208.40 ± 51.29 | 80.52 ± 37.25 | 79.61 ± 3.19 | 28.78 ± 12.53 | 15.11 ± 3.59 | 528.96 ± 362.28 | 1168.91 ± 807.44 |
| Confident | 1.68 ± .26 | 206.67 ± 50.3 | 78.71 ± 36.73 | 80.50 ± 3.48 | 28.35 ± 11.12 | 14.47 ± 3.61 | 571.18 ± 258.71 | 1294.10 ± 691.39 |
| Dominant | 1.80 ± .31 | 195.64 ± 43.25 | 68.06 ± 31.53 | 81.56 ± 3.41 | 29.42 ± 11.43 | 14.41 ± 3.45 | 592.32 ± 320.51 | 1316.36 ± 766.57 |
| Hostile | 1.74 ± .32 | 192.13 ± 38.75 | 75.72 ± 42.98 | 79.86 ± 4.63 | 31.24 ± 10.91 | 13.39 ± 3.23 | 594.37 ± 317.13 | 1445.72 ± 709.73 |
| Large | 2.08 ± .36 | 192.53 ± 51.6 | 70.09 ± 32.58 | 80.67 ± 4.14 | 27.97 ± 10.83 | 15.25 ± 3.61 | 508.46 ± 283.42 | 1060.69 ± 636.82 |
| Small | 1.95 ± .36 | 259.89 ± 95.63 | 86.36 ± 38.40 | 75.81 ± 3.8 | 32.89 ± 11.18 | 14.93 ± 3.7 | 504.28 ± 234.7 | 1138.36 ± 623.22 |
| *Note*. SD = standard deviation, HNR = Harmonics-to-noise-ratio. | | | | | | | | |

**S8.** **Acoustic description of vocal recordings.** Extraction of acoustic parameters was done using PRAAT (Boersma & Weenink, 2015). Table shows mean values and standard deviations.

References for Supplementary Materials

Boersma, P., & Weenink, D. (2015)*. Praat: Doing phonetics by computer. Version 6.0. 1*5.
